# Racial and Ethnic Inequities in Cancer Care Continuity During the COVID-19 Pandemic Among Those With SARS-CoV-2

**DOI:** 10.1001/jamanetworkopen.2024.12050

**Published:** 2024-05-20

**Authors:** Jessica Y. Islam, Cassandra A. Hathaway, Emma Hume, Kea Turner, Julie Hallanger-Johnson, Shelley S. Tworoger, Marlene Camacho-Rivera

**Affiliations:** 1 Center for Immunization and Infection Research in Cancer, H. Lee Moffitt Cancer Center and Research Institute, Tampa, Florida; 2 Department of Cancer Epidemiology, H. Lee Moffitt Cancer Center and Research Institute, Tampa, Florida; 3 Department of Health Outcomes and Behavior, H. Lee Moffitt Cancer Center and Research Institute, Tampa, Florida; 4 Department of Gastrointestinal Oncology, H. Lee Moffitt Cancer Center and Research Institute, Tampa, Florida; 5 Division of Endocrinology, Department of Medicine, Mayo Clinic, Rochester, Minnesota; 6 Division of Oncological Sciences, School of Medicine, Oregon Health & Science University, Portland; 7 Department of Community Health Sciences, School of Public Health, SUNY Downstate Health Sciences University, New York, New York

## Abstract

**Importance:**

Racially and ethnically minoritized US adults were disproportionately impacted by the COVID-19 pandemic and experience poorer cancer outcomes, including inequities in cancer treatment delivery.

**Objective:**

To evaluate racial and ethnic disparities in cancer treatment delays and discontinuations (TDDs) among patients with cancer and SARS-CoV-2 during different waves of the COVID-19 pandemic in the United States.

**Design, Setting, and Participants:**

This cross-sectional study used data from the American Society of Clinical Oncology Survey on COVID-19 in Oncology Registry (data collected from April 2020 to September 2022), including patients with cancer also diagnosed with SARS-CoV-2 during their care at 69 US practices. Racial and ethnic differences were examined during 5 different waves of the COVID-19 pandemic in the United States based on case surge (before July 2020, July to November 2020, December 2020 to March 2021, April 2021 to February 2022, and March to September 2022).

**Exposures:**

Race and ethnicity.

**Main Outcomes and Measures:**

TDD was defined as any cancer treatment postponed more than 2 weeks or cancelled with no plans to reschedule. To evaluate TDD associations with race and ethnicity, adjusted prevalence ratios (aPRs) were estimated using multivariable Poisson regression, accounting for nonindependence of patients within clinics, adjusting for age, sex, body mass index, comorbidities, cancer type, cancer extent, and SARS-CoV-2 severity (severe defined as death, hospitalization, intensive care unit admission, or mechanical ventilation).

**Results:**

A total of 4054 patients with cancer and SARS-CoV-2 were included (143 [3.5%] American Indian or Alaska Native, 176 [4.3%] Asian, 517 [12.8%] Black or African American, 469 [11.6%] Hispanic or Latinx, and 2747 [67.8%] White; 2403 [59.3%] female; 1419 [35.1%] aged 50-64 years; 1928 [47.7%] aged ≥65 years). The analysis focused on patients scheduled (at SARS-CoV-2 diagnosis) to receive drug-based therapy (3682 [90.8%]), radiation therapy (382 [9.4%]), surgery (218 [5.4%]), or transplant (30 [0.7%]), of whom 1853 (45.7%) experienced TDD. Throughout the pandemic, differences in racial and ethnic inequities based on case surge with overall TDD decreased over time. In multivariable analyses, non-Hispanic Black (third wave: aPR, 1.56; 95% CI, 1.31-1.85) and Hispanic or Latinx (third wave: aPR, 1.35; 95% CI, 1.13-1.62) patients with cancer were more likely to experience TDD compared with non-Hispanic White patients during the first year of the pandemic. By 2022, non-Hispanic Asian patients (aPR, 1.51; 95% CI, 1.08-2.12) were more likely to experience TDD compared with non-Hispanic White patients, and non-Hispanic American Indian or Alaska Native patients were less likely (aPR, 0.37; 95% CI, 0.16-0.89).

**Conclusions and Relevance:**

In this cross-sectional study of patients with cancer and SARS-CoV-2, racial and ethnic inequities existed in TDD throughout the pandemic; however, the disproportionate burden among racially and ethnically minoritized patients with cancer varied across SARS-CoV-2 waves. These inequities may lead to downstream adverse impacts on cancer mortality among minoritized adults in the United States.

## Key Points

**Question:**

Among patients with cancer and a confirmed SARS-CoV-2 infection identified during their cancer treatment planning, were there racial and ethnic differences in cancer treatment continuity throughout different waves of the COVID-19 pandemic?

**Findings:**

In this cross-sectional study of 4054 patients with cancer and SARS-CoV-2 infection, non-Hispanic Black and Hispanic or Latinx patients with cancer were more likely to experience cancer treatment delays or discontinuations (TDDs) compared with non-Hispanic White patients during the first year of the pandemic; by 2022, non-Hispanic Asian patients were more likely to experience cancer TDDs compared with non-Hispanic White patients, and non-Hispanic American Indian or Alaska Native patients were less likely.

**Meaning:**

Due to the delays or cancellations in cancer treatment observed in this study, downstream inequities in cancer outcomes among minoritized racial and ethnic groups may occur in the future and may differ across race and ethnicity due to differential impacts based on case surge during the pandemic.

## Teaser

This cross-sectional study examines disparities in cancer treatment delay or discontinuation among US patients with cancer and SARS-CoV-2 by race and ethnicity across 5 waves of the COVID-19 pandemic.

## Introduction

Societal disruptions due to the COVID-19 pandemic negatively impacted the continuity of care across the cancer continuum, including those undergoing cancer treatment.^1,2^ In response to the spread of SARS-CoV-2 infection and the growing number of COVID-19–related deaths in the United States, the US Centers for Disease Control and Prevention (CDC) implemented preventive guidelines to reduce risk of exposure to SARS-CoV-2 infection, such as stay-at-home orders during periods of peak community-level transmission prior to vaccine development.^3^ In response, oncology teams made adjustments to their cancer care plans, including rescheduling or postponing cancer screenings, shifting care to telehealth options, and delaying in-person procedures (ie, surgery).^2,4,5,6,7,8^ National cancer care guidelines were developed to provide guidance to cancer care teams, encouraging clinicians to weigh the risks and benefits of active cancer treatment in the context of COVID-19.^9^ Such adaptations to cancer treatment plans were particularly important given the elevated risk of COVID-19 acquisition and poor outcomes among patients with cancer.^10^ Modifications to operations within cancer care facilities and associated treatment plans for their patients led to downstream consequences,^11^ particularly disproportionately impacting populations at higher risk of poor COVID-19 outcomes.

Racial and ethnic minority groups were more likely to experience poor outcomes during the COVID-19 pandemic in the United States compared with their non-Hispanic White counterparts. For example, Black or African American adults and Hispanic or Latinx adults were 3 and 5 times more likely to develop COVID-19, respectively, and more likely to experience COVID-19–related hospitalization and mortality.^12,13,14,15,16^ Furthermore, Asian American adults had the highest risk of intensive care admission associated with COVID-19.^17^ Multilevel social determinants of health contributed to racial and ethnic inequities in COVID-19 outcomes, such as economic inequality, residential segregation, crowded living conditions, dependence on public transport, and higher work-related exposures.^17^ The disproportionate impact of COVID-19 on racial and ethnic minoritized communities also worsened preexisting disparities in cancer care delivery. During the COVID-19 pandemic, we observed that Black and Hispanic or Latinx patients were more likely to experience delays in care even after adjustment for COVID-19 disease severity.^18,19^ Factors that contributed to racial inequities in cancer treatment delays during the COVID-19 pandemic have not been explored, despite the potential role of case surges throughout the course of the pandemic in the United States. Insights into changes over time throughout the pandemic may inform future planning during public health emergencies and provide context for any downstream inequities in cancer mortality that may be observed on a population-level in the future. Our objective was to evaluate racial and ethnic inequities in cancer treatment delays or discontinuations (TDDs) over time during the US COVID-19 pandemic among patients with a SARS-COV-2 infection diagnosed during their treatment planning. We hypothesized that prevalence of cancer treatment delays would reduce over time across all racial and ethnic groups and that inequities in cancer treatment by race and ethnicity would change over time.

## Methods

### Data Source

We used the American Society of Clinical Oncology (ASCO) Survey on COVID-19 in Oncology Registry,^20^ which is a collaborative effort between ASCO and oncology practices across the United States to gather pertinent data on COVID-19 treatments and outcomes as well as cancer treatment and outcomes among patients with cancer who have been diagnosed with SARS-CoV-2 infection or COVID-19. Data for the present analysis were collected from April 2020 to September 2022 and include practice-reported information from a diverse sample of oncology care clinics, including private practices, practices that are part of health systems or hospitals, and academic practices. Sixty-nine practices participated in the ASCO registry, with 16 (23%) being academic institutions, 37 (54%) nonacademic hospitals or health systems, and 16 (23%) physician-owned or independent clinics. Additionally, of the 69 clinics, 22 (32%) were in the Midwest census region, 12 (17%) in the Northeast, 25 (36%) in the South, and 10 (15%) in the West. Further details regarding data collection procedures have been previously described.^20,21^ ASCO received approval from the Western Institutional Review Board to conduct the registry. The present study was reviewed by the Scientific Review Board at H. Lee Moffitt Cancer Center and was deemed non–human participants research as it does not meet the definition of human participant as defined in 45 CFR 46.102. Therefore, the requirement for informed consent was waived. The study was reported in accordance with the Strengthening the Reporting of Observational Studies in Epidemiology (STROBE) reporting guidelines.^22^

Participating oncology clinics included patients in the ASCO registry based on 2 eligibility criteria: (1) had a confirmed SARS-CoV-2 infection (requires testing verification, but does not capture test information) and (2) at the time of confirmed SARS-CoV-2 infection, was in 1 of the following 4 categories: (1) patient with a new cancer diagnosis and in the process of cancer staging and/or receipt of initial cancer therapy; (2) patient with clinically evident cancer receiving anticancer treatment; (3) patient who is cancer free and receiving any type of adjuvant therapy (including hormonal treatments) within 1 year after surgical resection (ie, patient is less than 1 year from having active cancer); or (4) patient with clinically evident cancer receiving supportive care only. Data were abstracted from each patient’s electronic health record and entered manually by clinic staff using a web-based REDCap survey project hosted on a secure server operated by ASCO. Practices entering the registry were able to submit patient data retrospectively for patients who met eligibility criteria and experienced SARS-CoV-2 infection prior to the clinic joining the registry.

### Measures

First, we assessed scheduled treatment at the time of infection, defined based on the following question: which of the following cancer treatment types was the patient receiving or scheduled to receive at the time of COVID-19 diagnosis? Clinics were able to select all that applied based on the following options: (1) surgery scheduled within 0 to 6 weeks after COVID-19 diagnosis; (2) radiation therapy; (3) drug-based therapy; (4) transplant; and (5) the patient was not receiving any of these anticancer therapies and had none planned at COVID-19 diagnosis. Patients for whom only option 5 was selected were excluded from this analysis (1303 of 5362 [24.3%]).

Our primary outcome was defined as cancer TDD. Cancer treatments considered were surgery, radiation therapy, drug-based therapies, and transplant (eg, bone marrow transplant) or cellular therapy (eg, CAR-T cell therapy). These treatments did not include those given while participating in a clinical trial. Treatments scheduled within 0 to 6 weeks after SARS-CoV-2 or COVID-19 diagnosis were included. TDD was based on the following question posed to clinics: “Which of the following describes how the patient’s treatment plan was modified at or immediately after COVID-19 diagnosis?” Answer options included: patient received on schedule or within 14 days, patient receipt of therapy or surgery was delayed at least 14 days from initial treatment date, or patient receipt of therapy or surgery was discontinued or canceled with no plans of restart. If a patient was scheduled for multiple treatment types, clinics provided a response about the treatment plan for each form of therapy. We defined TDD as those who experienced treatment delays at least 14 days from initially scheduled treatment date or a discontinuation of treatment for at least 1 of the indicated treatments included in the patient’s treatment plan.

The main exposures included race and ethnicity as a proxy measure for the racialized experiences of patients with cancer during the pandemic, as documented within each patient’s electronic health record^23,24^ and COVID-19 case surge waves. To account for COVID-19 case surges^3,25,26^ during the pandemic that may have affected TDD, we created a time variable based on the patient’s SARS-CoV-2 diagnosis date as follows: first wave, from April to June 2020; second wave, from July to November 2020; third wave, from December 2020 to March 2021 (Alpha variant dominant); fourth wave, from April 2021 to February 2022 (Delta variant dominant); and fifth wave from March to September 2022 (Omicron variant dominant). Racial and ethnic categories were recorded using the following categories: Hispanic or Latinx, non-Hispanic American Indian or Alaska Native, non-Hispanic Asian, non-Hispanic Black or African American, and non-Hispanic White. Patient race and ethnicity was recorded for all participants, with no missing values, and patients were categorized into 1 racial and ethnic category within the ASCO registry. Documented patient demographic and clinical information included age category at SARS-CoV-2 infection diagnosis, sex, rurality of patient’s residence, census region of patient, tobacco use history (current, former, never, or unsure), body mass index (BMI; calculated as weight in kilograms divided by height in meters squared), number of comorbidities, and Eastern Cooperative Oncology Group performance status scale. Cancer clinical information collected included cancer type using *International Statistical Classification of Diseases and Related Health Problems, Tenth Revision *(*ICD-10*) codes, extent of cancer (local, regional, metastatic, or cancer free but receiving adjuvant therapy), cancer status (progressing, stable, unknown, or responding to treatment), and scheduled treatment at the time of COVID-19 infection diagnosis. COVID-19 severity was defined based on a patient’s most severe reported disease status, including mechanical ventilation, hospitalization, intensive care unit (ICU) admission, and death due to COVID-19 within 30 days of diagnosis. We included patients with cancer who died due to COVID-19 because the decision to discontinue or delay cancer treatment occurred at SARS-CoV-2 diagnosis (ie, prior to death). Only patients with planned cancer treatment at the time of SARS-CoV-2 infection diagnosis were included in the present study.

### Statistical Analysis

We summarized patient characteristics and cancer treatment plans at COVID-19 diagnosis as percentages by COVID-19 case surge periods (ie, waves) overall and by race and ethnicity and compared them using Pearson χ^2^ or exact tests. We computed prevalence ratios with multivariable Poisson regression using robust estimation of standard errors^27,28,29^ to evaluate the associations of race and ethnicity with experiencing any TDD by COVID-19 case surge wave. We accounted for nonindependence of patients within clusters at the facility level via calculating cluster-robust standard errors. Using directed acyclic graphs as our analytic framework, multivariable models were adjusted for age, sex, BMI, number of comorbidities, cancer type, cancer extent, and COVID-19 diagnosis severity (death, hospitalization, ICU admission, or mechanical ventilation). We assessed each covariate for collinearity with all other covariates and used a complete case approach. Based on the exploratory and descriptive nature of this analysis, we did not include an adjustment for multiple comparisons for data presentation.^30^ Statistical significance was defined as a 2-sided *P* < .05. All analyses were performed with Stata version 15.1 (StataCorp).

## Results

### Sample Characteristics

Overall, 4054 patients with cancer and SARS-CoV-2 infection were recorded in the ASCO registry with scheduled anticancer therapy at the time of infection, including patients mostly older than 50 years (1419 [35.1%] aged 50-64 years; 1928 [47.7%] aged ≥65 years), 2403 (59.3%) female patients, and 3436 (84.8%) residing in urban areas (Table 1). Overall, 143 (3.5%) were non-Hispanic American Indian or Alaska Native adults, 176 (4.3%) were non-Hispanic Asian adults, 517 (12.8%) were non-Hispanic Black or African American adults, 469 (11.6%) were Hispanic or Latinx adults, and 2747 (67.8%) were non-Hispanic White adults. Race and ethnicity was missing for only 2 patients in the ASCO registry. Approximately three-quarters of patients were diagnosed with a solid tumor, and approximately one-third had metastatic disease at the time of SARS-CoV-2 diagnosis. The most common primary cancers included breast cancer (1108 [27.3%]); lymphomas, leukemias, myeloma (950 [23.4%]); lung cancer (418 [10.3%]); and gastrointestinal cancers (472 [11.6%]). At the time of SARS-CoV-2 detection, approximately 90% of patients (3682 [90.8%]) were scheduled for drug-based therapies (eg, chemotherapy), 5.4% (218) were scheduled for surgery, and 9.4% (382) were scheduled for radiation therapy.

**Table 1.** Sociodemographic Characteristics of Patients With Cancer and SARS-CoV-2 Infection During Treatment Planning Captured in the American Society of Clinical Oncology Cancer and COVID-19 Registry, Stratified by COVID-19 Case Surge Wave

Characteristics	Patients, No. (%)						*P* value
	Total (N = 4054)	First wave (n = 202)	Second wave (n = 798)	Third wave (n = 1036)	Forth wave (n = 1389)	Fifth wave (n = 629)	
Age at SARS-CoV-2 infection diagnosis (4039 patients with data), y^a^							
18-34	142 (3.5)	8 (4.0)	41 (5.1)	27 (2.6)	43 (3.1)	23 (3.7)	<.001
35-49	550 (13.6)	44 (22.0)	110 (13.8)	124 (12.0)	182 (13.1)	90 (14.6)	
50-64	1419 (35.1)	53 (26.5)	282 (35.3)	379 (36.6)	507 (36.5)	198 (32.1)	
≥65	1928 (47.7)	95 (47.5)	365 (45.7)	506 (48.8)	656 (47.3)	306 (49.6)	
Sex							
Male	1651 (40.7)	91 (45.0)	339 (42.5)	399 (38.5)	567 (40.8)	255 (40.5)	.32
Female	2403 (59.3)	111 (55.0)	459 (57.5)	637 (61.5)	822 (59.2)	374 (59.5)	
Race and ethnicity (4052 patients with data)^a^							
Hispanic/Latinx	469 (11.6)	34 (16.8)	91 (11.4)	71 (6.9)	127 (9.1)	146 (23.2)	<.001
Non-Hispanic American Indian/Alaskan Native	143 (3.5)	9 (4.5)	30 (3.8)	35 (3.4)	47 (3.4)	22 (3.5)	
Non-Hispanic Asian	176 (4.3)	23 (11.4)	29 (3.6)	47 (4.5)	48 (3.5)	29 (4.6)	
Non-Hispanic Black or African American	517 (12.8)	37 (18.3)	152 (19.1)	113 (10.9)	157 (11.3)	58 (9.2)	
Non-Hispanic White	2747 (67.8)	99 (49.0)	494 (62.1)	770 (74.3)	1010 (72.7)	374 (59.5)	
Rurality of patient’s residence (4053 patients with data)^a^							
Urban	3436 (84.8)	193 (95.5)	676 (84.7)	876 (84.6)	1132 (81.5)	559 (88.9)	<.001
Rural	617 (15.2)	9 (4.5)	122 (15.3)	159 (15.4)	257 (18.5)	70 (11.1)	
Census region (4053 patients with data)^a^							
Midwest	1284 (31.7)	27 (13.4)	223 (27.9)	313 (30.2)	553 (39.8)	168 (26.7)	<.001
Northeast	563 (13.9)	117 (57.9)	189 (23.7)	138 (13.3)	100 (7.2)	19 (3.0)	
South	1875 (46.3)	40 (19.8)	291 (36.5)	532 (51.4)	632 (45.5)	380 (60.4)	
West	331 (8.2)	18 (8.9)	95 (11.9)	52 (5.0)	104 (7.5)	62 (9.9)	
Tobacco use							
Current smoker	370 (9.1)	15 (7.4)	55 (6.9)	87 (8.4)	158 (11.4)	55 (8.7)	<.001
Former smoker	1541 (38.0)	64 (31.7)	283 (35.5)	420 (40.5)	539 (38.8)	235 (37.4)	
Never smoked	2014 (49.7)	94 (46.5)	424 (53.1)	508 (49.0)	658 (47.4)	330 (52.5)	
Unsure	129 (3.2)	29 (14.4)	36 (4.5)	21 (2.0)	34 (2.4)	9 (1.4)	
BMI (3944 patients with data)^a^							
Underweight (<18.5)	92 (2.3)	8 (4.1)	17 (2.2)	15 (1.5)	37 (2.7)	15 (2.5)	.05
Healthy weight (18.5-24.9)	1040 (26.4)	56 (28.9)	213 (27.6)	259 (25.6)	343 (25.1)	169 (28.0)	
Overweight (25.0-29.9)	1249 (31.7)	70 (36.1)	259 (33.5)	322 (31.9)	410 (30.0)	188 (31.2)	
Obesity (≥30.0)	1563 (39.6)	60 (30.9)	283 (36.7)	414 (41.0)	575 (42.1)	231 (38.3)	
Comorbidities, No.^b^							
0	1498 (37.0)	63 (31.2)	255 (32.0)	406 (39.2)	530 (38.2)	244 (38.8)	<.001
1-2	1973 (48.7)	94 (46.5)	392 (49.1)	510 (49.2)	671 (48.3)	306 (48.6)	
3-4	441 (10.9)	29 (14.4)	70 (8.8)	101 (9.7)	171 (12.3)	70 (11.1)	
≥5	142 (3.5)	16 (7.9)	81 (10.2)	19 (1.8)	17 (1.2)	9 (1.4)	
Cancer information							
Type							
Breast	1108 (27.3)	37 (18.3)	181 (22.7)	327 (31.6)	402 (28.9)	161 (25.6)	<.001
Lung	418 (10.3)	20 (9.9)	91 (11.4)	101 (9.7)	149 (10.7)	57 (9.1)	
Genitourinary	362 (8.9)	21 (10.4)	74 (9.3)	94 (9.1)	118 (8.5)	55 (8.7)	
Gastrointestinal	472 (11.6)	20 (9.9)	88 (11.0)	143 (13.8)	148 (10.7)	73 (11.6)	
Gynecological	231 (5.7)	18 (8.9)	50 (6.3)	46 (4.4)	75 (5.4)	42 (6.7)	
Leukemia, lymphomas, and myeloma	950 (23.4)	58 (28.7)	192 (24.1)	219 (21.1)	342 (24.6)	139 (22.1)	
Other solid tumors	513 (12.7)	28 (13.9)	122 (15.3)	106 (10.2)	155 (11.2)	102 (16.2)	
Cancer diagnosis year (4052 patients with data)^a^							
2010 or earlier	218 (5.4)	16 (7.9)	60 (7.5)	59 (5.7)	59 (4.2)	24 (3.8)	<.001
2011-2019	1863 (46.0)	129 (63.9)	462 (58.0)	513 (49.5)	558 (40.2)	201 (32.0)	
2020-2022	1971 (48.6)	57 (28.2)	274 (34.4)	464 (44.8)	772 (55.6)	404 (64.2)	
Extent of cancer							
Local	828 (20.4)	35 (17.3)	180 (22.6)	210 (20.3)	264 (19.0)	139 (22.1)	<.001
Regional	426 (10.5)	13 (6.4)	67 (8.4)	127 (12.3)	169 (12.2)	50 (7.9)	
Metastatic	1521 (37.5)	75 (37.1)	328 (41.1)	387 (37.4)	509 (36.6)	222 (35.3)	
Cancer free but receiving adjuvant therapy	228 (5.6)	10 (5.0)	24 (3.0)	84 (8.1)	73 (5.3)	37 (5.9)	
Unknown	1051 (25.9)	69 (34.2)	199 (24.9)	228 (22.0)	374 (26.9)	181 (28.8)	
ECOG performance score (3077 patients with data)^a^							
0	1295 (42.1)	43 (31.2)	216 (41.0)	340 (41.0)	452 (41.7)	244 (49.0)	<.001
1	1239 (40.3)	49 (35.5)	204 (38.7)	353 (42.6)	449 (41.4)	184 (36.9)	
2	396 (12.9)	28 (20.3)	70 (13.3)	100 (12.1)	138 (12.7)	60 (12.0)	
≥3	147 (4.8)	18 (13.0)	37 (7.0)	36 (4.3)	46 (4.2)	10 (2.0)	
Cancer status at the time of COVID-19 diagnosis							
Progressing	559 (13.8)	45 (22.3)	124 (15.5)	132 (12.7)	196 (14.1)	62 (9.9)	<.001
Stable	1249 (30.8)	56 (27.7)	322 (40.4)	382 (36.9)	340 (24.5)	149 (23.7)	
Unknown	454 (11.2)	14 (6.9)	78 (9.8)	93 (9.0)	167 (12.0)	102 (16.2)	
Responding to treatment	276 (6.8)	5 (2.5)	1 (0.1)	69 (6.7)	142 (10.2)	59 (9.4)	
Unknown	1516 (37.4)	82 (40.6)	273 (34.2)	360 (34.7)	544 (39.2)	257 (40.9)	
Surgery to resect or remove cancer within 6 wk prior to COVID-19 diagnosis							
No	3773 (93.1)	191 (94.6)	753 (94.4)	980 (94.6)	1275 (91.8)	574 (91.3)	.001
Yes	95 (2.3)	3 (1.5)	19 (2.4)	26 (2.5)	38 (2.7)	9 (1.4)	
Unknown	186 (4.6)	8 (4.0)	26 (3.3)	30 (2.9)	76 (5.5)	46 (7.3)	
Scheduled treatment at the time of COVID-19 diagnosis^c^							
Surgery scheduled within 0-6 wk after COVID-19 diagnosis	218 (5.4)	8 (4.0)	51 (6.4)	43 (4.2)	75 (5.4)	41 (6.5)	.13
Radiation therapy	382 (9.4)	12 (5.9)	77 (9.6)	99 (9.6)	143 (10.3)	51 (8.1)	.24
Drug-based therapy	3682 (90.8)	189 (93.6)	707 (88.6)	963 (93.0)	1254 (90.3)	569 (90.5)	.01
Transplant	30 (0.7)	0	17 (2.1)	3 (0.3)	6 (0.4)	4 (0.6)	<.001
Cancer treatment delay among those with scheduled treatment							
No delays with treatment scheduled or within 14 d of original date	2201 (54.3)	68 (33.7)	343 (43.0)	568 (54.8)	806 (58.0)	416 (66.1)	<.001
Scheduled cancer treatment delayed at least 14 d	1509 (37.2)	81 (40.1)	359 (45.0)	386 (37.3)	499 (35.9)	184 (29.3)	
Scheduled cancer treatment was discontinued or canceled with no plans of restart	344 (8.5)	53 (26.2)	96 (12.0)	82 (7.9)	84 (6.0)	29 (4.6)	
COVID-19 information							
Patient vaccinated for COVID-19 (before or after COVID-19 diagnosis) (2727 patients with data)^a^							
No	1014 (37.2)	95 (99.0)	7 (100)	377 (56.3)	395 (29.5)	140 (22.7)	<.001
Yes	1048 (38.4)	0	0	32 (4.8)	584 (43.7)	432 (70.0)	
Unsure	665 (24.4)	1 (1.0)	0	261 (39)	358 (26.8)	45 (7.3)	
COVID-19 diagnosis severity^d^							
Uncomplicated	2784 (68.7)	79 (39.1)	516 (64.7)	711 (68.6)	973 (70.1)	505 (80.3)	<.001
Hospitalized	892 (22.0)	66 (32.7)	197 (24.7)	245 (23.6)	295 (21.2)	89 (14.1)	
ICU admission	90 (2.2)	4 (2.0)	18 (2.3)	23 (2.2)	38 (2.7)	7 (1.1)	
Mechanically ventilated	46 (1.1)	8 (4.0)	18 (2.3)	4 (0.4)	11 (0.8)	5 (0.8)	
Death within 30 d of COVID-19	242 (6.0)	45 (22.3)	49 (6.1)	53 (5.1)	72 (5.2)	23 (3.7)	
Patient developed any COVID-19 complications							
No	3259 (80.4)	102 (50.5)	610 (76.4)	858 (82.8)	1140 (82.1)	549 (87.3)	<.001
Yes	795 (19.6)	100 (49.5)	188 (23.6)	178 (17.2)	249 (17.9)	80 (12.7)	
Types of COVID-19 complications (1258 patients with data)							
Systemic complications	76 (9.6)	25 (25.0)	14 (7.4)	10 (5.6)	15 (6.0)	12 (15.0)	<.001
Pulmonary complications	399 (50.2)	22 (22.0)	80 (42.6)	100 (56.2)	151 (60.6)	46 (57.5)	
Cardiovascular complications	125 (15.7)	13 (13.0)	34 (18.1)	23 (12.9)	46 (18.5)	9 (11.3)	
Gastrointestinal complications	11 (1.4)	3 (3.0)	2 (1.1)	4 (2.2)	1 (0.4)	1 (1.3)	
Other complications	184 (23.1)	37 (37.0)	58 (30.9)	41 (23.0)	36 (14.5)	12 (15.0)	

Abbreviations: BMI, body mass index (calculated as weight in kilograms divided by height in meters squared); ECOG, Eastern Cooperative Oncology Group; ICU, intensive care unit.

^a^ Missing data: age (n = 15); race and ethnicity (n = 2); rurality (n = 1); census region (n = 1); BMI (n = 110); ECOG (n = 977); surgery (n = 186); COVID-19 vaccination (n = 1327).

^b^ Potential comorbidities include alcoholism, chronic supplemental oxygen needed, cirrhosis, congestive heart failure, coronary artery disease, dementia, diabetes, hepatitis, history of solid organ transplant, HIV/AIDS, hypertension, immunosuppressed due to non–cancer-related treatment (defined as outpatient use of systemic corticosteroids [≥10mg/d prednisone], use of chemotherapy, use of immunosuppressive agents for solid organ transplant or for an autoimmune disease), inflammatory bowel disease, pulmonary disease, kidney disease, systemic autoimmune disease.

^c^ Multiple scheduled treatments possible.

^d^ Severe COVID-19 was defined as either death due to COVID-19, hospitalization, ventilator use, or ICU admission (any combination).

### Cancer TDD

**Figure 1.**
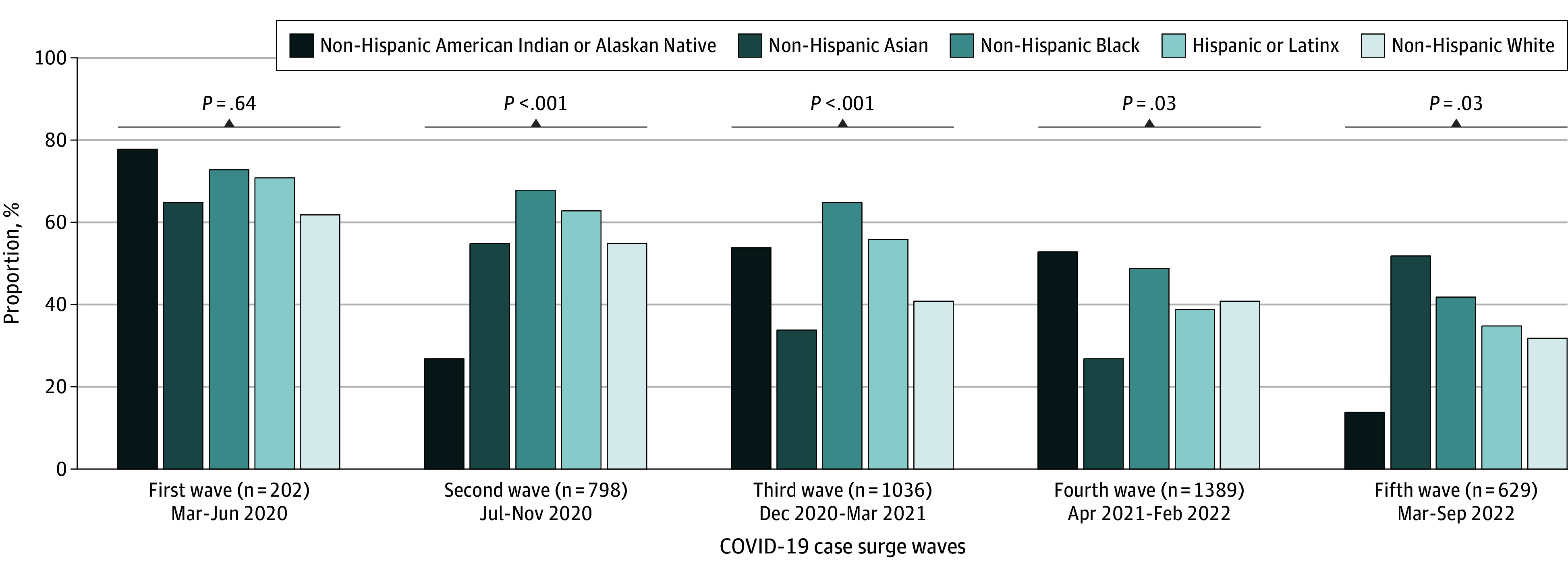
Prevalence of Cancer Treatment Delays (≥14 Days) or Discontinuations by Race and Ethnicity Among Patients With Cancer Diagnosed With SARS-CoV-2 During the COVID-19 Pandemic, American Society of Clinical Oncology COVID-19 and Cancer Registry Participants

Overall, among patients with cancer with scheduled cancer treatment at the time of SARS-CoV-2 diagnosis, 1853 (45.7%) experienced a treatment delay (≥14 days) or discontinuation. We observed that TDDs decreased, with 66.3% of patients (134 of 202) experiencing a TDD during the first wave of the pandemic, decreasing to 57.0% (455 of 798), 45.2% (468 of 1036), 41.9% (583 of 1389), and 33.9% (213 of 629) in subsequent surges of COVID cases (*P* < .001, Table 1). Figure 1 summarizes the prevalence of TDD by race and ethnicity overall and stratified by COVID-19 cases surge, demonstrating that the prevalence of cancer TDDs reduced over time throughout the pandemic across racial and ethnic groups. Table 2 summarizes the reasons for cancer TDD from the clinic perspective by COVID-19 pandemic case wave and treatment type. Across treatment modality, the most common cause of cancer TDD by pandemic wave was the patient’s COVID-19 disease.

**Table 2.** Primary Reasons for Delay or Discontinuation of Scheduled Cancer Treatment Among Patients With Cancer Diagnosed With SARS-CoV-2 Throughout the COVID-19 Pandemic

Reason^a^	Patients, No./total No. (%)					
	Total	First wave	Second wave	Third wave	Fourth wave	Fifth wave
**Primary reason for the delay or discontinuation of one or more drug-based agents**						
Progressive or recurrent disease	52/1584 (3.3)	10/124 (8.1)	12/384 (3.1)	8/405 (2)	13/498 (2.6)	9/173 (5.2)
Treatment-related toxic effects	56/1584 (3.5)	1/124 (0.8)	13/384 (3.4)	14/405 (3.5)	22/498 (4.4)	6/173 (3.5)
Patient’s COVID-19 disease	1325/1584 (83.6)	104/124 (83.9)	312/384 (81.3)	349/405 (86.2)	429/498 (86.1)	131/173 (75.7)
Lack of clinical resources	3/1584 (0.2)	0	2/384 (0.5)	0	1/498 (0.2)	0
Patient’s choice	35/1584 (2.2)	3/124 (2.4)	16/384 (4.2)	6/405 (1.5)	6/498 (1.2)	4/173 (2.3)
Other or unknown	113/1584 (7.1)	6/124 (4.8)	29/384 (7.6)	28/405 (6.9)	27/498 (5.4)	23/173 (13.3)
**Primary reason for the delay or cancellation of surgery?**						
Progressive or recurrent disease	3/133 (2.3)	0	1/33 (3.0)	0	2/43 (4.7)	0
Patient’s COVID-19 disease	125/133 (94.0)	6/6 (100)	32/33 (97.0)	31/33 (93.9)	40/43 (93.0)	16/18 (88.9)
Other or unknown	5/133 (3.8)	0	0	2/33 (6.1)	1/43 (2.3)	2/18 (11.1)
**Primary reason for the delay, alteration, or discontinuation of radiation therapy?**						
Progressive or recurrent disease	5/159 (3.1)	0	2/46 (4.3)	0	3/51 (5.9)	0
Patient’s COVID-19 disease	133/159 (83.6)	5/5 (100)	38/46 (82.6)	45/50 (90.0)	41/51 (80.4)	4/7 (57.1)
Patient’s choice	6/159 (3.8)	0	3/46 (6.5)	1/50 (2.0)	0	2/7 (28.6)
Other or unknown	15/159 (9.4)	0	3/46 (6.5)	4/50 (8.0)	7/51 (13.7)	1/7 (14.3)
**Primary reason for the delay or discontinuation of transplant or cellular therapy?**						
Patient’s COVID-19 disease	19/23 (82.6)	0	12/15 (80.0)	3 (100)	4 (100)	0
Lack of clinical resources	1/23 (4.3)	0	1/15 (6.7)	0	0	0
Other or unknown	3/23 (13)	0	2/15 (13.3)	0	0	1 (100)

^a^ Missing data: drug based (2098 patients); surgery (85 patients); radiation (223 patients); transplant (7 patients).

Furthermore, we examined cancer treatment discontinuations with no plans of restart and observed the prevalence was 8.5% (344 patients). By race and ethnicity, non-Hispanic Asian patients had the highest prevalence of cancer treatment discontinuations at 13.1% (23 patients), followed by non-Hispanic Black or African American patients at 12.8% (66 patients), non-Hispanic White patients at 7.7% (212 patients), and non-Hispanic American Indian or Alaska Native patients at 6.9% (10 patients) (data not shown). When examined by COVID-19 pandemic waves, cancer treatment discontinuations also significantly decreased across time, with the prevalence during the first wave at 26.2% (53 patients), 12.0% during the second wave (96 patients), 7.9% during the third wave (82 patients), 6.1% during the fourth wave (84 patients), and 4.6% during the final wave (29 patients) (*P* < .001).

**Figure 2.**
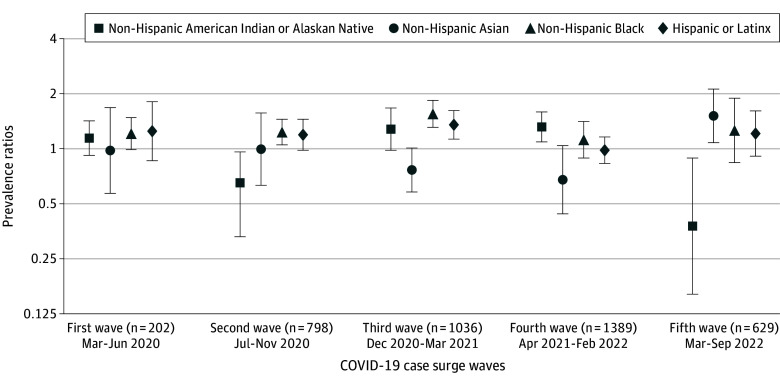
Racial and Ethnic Inequities of Any Cancer Treatment Delays (≥14 Days) or Discontinuations Among Patients With Cancer Diagnosed With SARS-CoV-2 During the COVID-19 Pandemic, American Society of Clinical Oncology COVID-19 and Cancer Registry Participants

Figure 2 summarizes the results of the multivariable analyses of racial and ethnic differences in cancer TDDs across surges of COVID-19 cases. During the first few months of the pandemic, inequities by race and ethnicity in cancer TDD were not observed. By the second wave, July to November 2020, we observed that non-Hispanic Black or African American patients with SARS-CoV-2 infection had 23% higher likelihood of experiencing a cancer TDD compared with non-Hispanic White patients (adjusted prevalence ratio [aPR], 1.23; 95% CI, 1.05-1.45). In contrast, non-Hispanic American Indian or Alaska Native patients were less likely than non-Hispanic White patients to experience a cancer TDD during the same period (aPR, 0.56; 95% CI, 0.33-0.96). By the third wave, during the rise of Alpha variant as the dominant strain, between December 2020 to March 2021, non-Hispanic Black or African American (aPR, 1.56; 95% CI, 1.31-1.84) and Hispanic or Latinx (aPR, 1.35; 95% CI, 1.13-1.62) patients were more likely to experience cancer TDD compared with non-Hispanic White patients with SARS-CoV-2 infection. During the fourth wave, between April 2021 and February 2022, when Delta was the dominant variant, we observed that non-Hispanic American Indian or Alaska Native patients with cancer were more likely to experience cancer TDD compared with their non-Hispanic White counterparts (aPR, 1.32; 95% CI, 1.09-1.59). During the last wave of the COVID-19 pandemic in 2022, when Omicron was the dominant variant, we observed that non-Hispanic Asian adults were more likely to experience a cancer TDD (aPR, 1.51; 95% CI, 1.08-2.12) and non-Hispanic American Indian or Alaska Native were less likely (aPR, 0.37; 95% CI, 0.16-0.89) compared with their non-Hispanic White counterparts.

## Discussion

In this investigation of racial and ethnic disparities in cancer TDD over time during the COVID-19 pandemic, we found that non-Hispanic Black or African American and Hispanic or Latinx patients with cancer and SARS-CoV-2 infection were more likely to experience treatment disruptions compared with their non-Hispanic White counterparts during the first year of the pandemic. During later months of the pandemic in 2022, we observed that non-Hispanic Asian patients with cancer had the highest prevalence of cancer TDD. Cancer TDD among American Indian or Alaska Native patients with cancer varied over time throughout the pandemic. Variations in the experiences of cancer TDD by racial and ethnic group throughout the pandemic is likely multifactorial, stemming from variations in state- or county-level policies affecting hospital and clinic workflows, COVID-19 vaccination uptake, trust in the health care system, and personal choices of the patients to undergo treatment during the ongoing pandemic. From the clinic perspective, across all types of scheduled cancer treatment, the most common reason for treatment delays was the patient’s COVID-19 disease. To our knowledge, this is the first analysis to comprehensively explore racial disparities in cancer treatment disruptions among patients with cancer and SARS-CoV-2 infection during different waves of the COVID-19 pandemic. Our findings are consistent with prior work that demonstrates racial and ethnic differences in timely receipt of cancer care and significant cancer treatment delays among patients with cancer in general (without COVID-19 disease).^31^ Our study suggests COVID-19 case surge played a substantial role in racial and ethnic disparities in cancer care treatment delivery and underscores the need to develop tailored interventions to ensure equitable cancer care delivery during public health emergencies.

The COVID-19 pandemic was characterized by periodic case surges leading to changes in precautionary measures enforced by various entities, including at the national, state, and hospital level based on the local prevalence of cases or deaths. It is important to contextualize our results based on case surges that occurred throughout the pandemic. Our observation period began in April 2020, which is when all US states and territories had declared emergency and disaster conditions due to increasing case counts and deaths associated with COVID-19. By the end of April, the total number of confirmed cases across the country surpassed 1 million and the US death toll became the highest in the world.^32^ During this first wave, while we did not observe racial inequities in cancer TDD, 66% of patients overall experienced a delay in treatment, with a range of 62% among non-Hispanic White adults to as high as 78% among non-Hispanic American Indian or Alaska Native adults. The high prevalence of cancer TDDs we observed during the early months of the pandemic is reflective of what has been previously reported.^7^ Throughout the summer of 2020 and early 2021, as the Alpha variant started to surge, we observed significant racial and ethnic inequities in cancer TDD among non-Hispanic Black or African American and Hispanic or Latinx patients. These racial inequities may be in part attributable to case surges, which disproportionately affected minoritized communities,^33,34^ that occurred throughout the remainder of 2020. For example, by the end of 2020, 2 case surges had occurred, with the third surge of cases leading to a total of 20 million cases by early 2021,^32,35^ representing an increase of more than 10 million cases in less than 2 months, which was likely attributable to the highly contagious Alpha variant of SARS-CoV-2 infection. A survey conducted among patients with cancer (without SARS-CoV-2), which also demonstrated Black and Hispanic or Latinx patients with cancer were more likely to experience a treatment delay compared with non-Hispanic White patients, found that Black respondents with cancer were most likely to feel extremely concerned about the pandemic affecting their cancer outcomes, their overall health, and extremely concerned about contracting COVID-19.^31^ Furthermore, Black or African American and Hispanic or Latinx patients with cancer were more likely to experience job loss, food insecurity, and loss of childcare during the pandemic compared to non-Hispanic White patients with cancer.

During the later waves of case surges during the pandemic attributable to the Delta and Omicron variants, we no longer observed significant differences in cancer TDD among non-Hispanic Black or African American and Hispanic or Latinx patients with cancer. However, inequities started to emerge among patients from other racially and ethnically minoritized groups. Asian patients with cancer and SARS-CoV-2 infection were most likely to experience cancer TDD compared with their non-Hispanic White counterparts by 2022 and the end of our observation period. Asian American individuals were profoundly impacted by the COVID-19 pandemic in ways that may have interfered with access to cancer care. First, an important consequence of the pandemic was an increase in xenophobia and anti-Asian sentiment and associated targeted crimes in the United States, which had adverse impacts on health care-seeking behaviors, particularly among older Asian individuals.^36,37,38^ Second, particularly relevant to our study setting, Asian Americans had the highest US COVID-19 hospitalization and mortality rates compared with other racial groups.^39,40^ Finally, Asian Americans led the nation in long-term COVID-19–related unemployment given the overrepresentation of essential workers at both ends of the socioeconomic or occupational spectrum from US health care workers to low-wage, no-benefits, front-line workers.^41^ While we only observed inequities in comparison with non-Hispanic White patients later in the pandemic, it is noteworthy that non-Hispanic Asian patients in our study population had cancer TDD prevalence rates as high as 65% and 55% during the first year of the pandemic. While significant inequities only arose for Asian patients in 2022, we observed variations in cancer TDD throughout the pandemic among non-Hispanic American Indian or Alaska Native populations, with this population less likely to experience cancer TDD by the end of our study period in 2022. It is well established that American Indian or Alaska Native populations in the United States have the highest COVID-19 vaccination rates compared with other groups, which may contribute to their willingness or ability to receive timely cancer treatment.^42^ However, prior work among American Indian or Alaska Native cancer survivors demonstrated that these populations experienced significant barriers to health care appointments during the pandemic with those who adhered to COVID-19 preventive behaviors and were as much as 6 times more likely to experience a delay in medical care compared with poorly adherent populations.^43^ Additional reasons for potential variations in cancer TDD among American and Alaskan Native patients with SARS-CoV-2 throughout the pandemic should be explored though qualitative investigations.

Delays in cancer care may incur significant detrimental impacts on cancer-related survival,^44^ which we will likely observe in the coming years. In the general population, the COVID-19 pandemic resulted in delays or cancellations in cancer-related diagnostic, clinical, and treatment activities, including screenings, surgery, radiotherapy, and outpatient visits.^45^ The long-term effects of these changes in cancer care are still to be delineated. Analyses using UK data from observational studies conducted between 2013 to 2017 modeled cancer progression and loss of life-years due to pandemic-related delays in surgical intervention. These analyses demonstrate that an estimated 3- or 6-month delay of surgery across all stage cancers may lead to an excess of 4755 and 10 760 deaths, respectively, among the 94 912 patients undergoing resections for major cancers annually.^46^ Similarly, a meta-analysis of studies estimating the impact of cancer treatment delays on mortality found that even a 4-week delay in cancer treatment can lead to a 6% to 8% increase in the risk of death across surgical, systemic treatment, and radiotherapy for several cancers.^44^ Early in the pandemic, US surgical oncologists and multidisciplinary cancer treatment teams were making clinical decisions regarding surgical interventions based on alternative nonsurgical treatment options, as recommended by the American College of Surgeons “Roadmap for Maintaining Essential Surgery during COVID-19 Pandemic.”^47^ However, the potential for uncertainty and subjectivity in clinical decision-making in the context of historical inequities in cancer treatment in the United States by race and ethnicity may magnify existing inequalities. Future research should focus on evaluating the long-term adverse impacts that the pandemic overall and comorbid SARS-CoV-2 infection had on survival among patients with cancer by racial and ethnic groups to further demonstrate the importance of keeping equity at the forefront of policy-level decision-making.

### Limitations

The results of our analysis should be interpreted within the context of several limitations. First, an important limitation of our study is that our data are limited to the clinic or oncology practice perspective regarding reasons for treatment delays. The patient perspective is vital to understanding reasons for cancer care disruptions during the pandemic. Future qualitative research should be conducted to document the experiences of patients with cancer diagnosed with SARS-CoV-2 and assess the patient-clinician communication experiences to contextualize the perception of patients regarding the risks and benefits of delaying cancer treatment during the pandemic. Second, given how closely tied employment is with insurance status in the United States,^41^ an important limitation of our study is that we did not have details on patient’s insurance type or whether patients were uninsured. The pandemic or a diagnosis of cancer may have led to disruptions in employment and associated insurance, which disproportionately impacts racially and ethnically minoritized populations.^48^ In the context of cancer treatment delays during the pandemic, it is important to consider insurance status as health care access factor to disentangle impacts of a COVID-19 diagnosis or employment loss on treatment disruptions. Third, among those who received drug-based therapy, we were unable to evaluate different types of therapy, such as intravenous (IV) chemotherapy vs oral chemotherapy, which is important in the context of treatment delays given the need for physical visits to obtain IV chemotherapy. Additionally, as the study only enrolled patients with a confirmed SARS-CoV-2 infection, we were unable to compare with patients with cancer without COVID-19. Future observational studies may explore differences in inequities over time among patients with and without COVID-19 to further disentangle the role of comorbid COVID-19 disease in exacerbating inequities.

## Conclusions

In this cross-sectional study of patients with cancer and SARS-CoV-2 infection, we observed important racial and ethnic disparities in cancer TDDs throughout different waves of the pandemic, hallmarking the importance of continuing to monitor the potential adverse downstream effects of the pandemic on cancer outcomes in the United States. Through this analysis, we provide important insights into the potential long-term impacts of the COVID-19 pandemic on cancer-specific outcomes. In the wake of the pandemic, it is important for oncology clinicians to engage in discussions with their patients, particularly patients from racially and ethnically minoritized communities, to ensure they receive the support they may need in the face of an increasing disproportionate burden of poor cancer outcomes in the coming years. Evaluating the downstream effects of clinician-level decisions on cancer treatment delivery in response to COVID-19 national policies will be an important area of research to delineate any adverse cancer-related outcomes, such as worsened survival.

## Notes

Supplement.
Data Sharing Statement
