# Supplementary material for: Racial and Ethnic Inequities in Cancer Care Continuity During the COVID-19 Pandemic Among Those With SARS-CoV-2
Source: JAMA Netw Open. 2024 May 20;7(5):e2412050. doi: 10.1001/jamanetworkopen.2024.12050 (PMC11107297; doi:10.1001/jamanetworkopen.2024.12050)
Supplement: Supplement. — Data Sharing Statement [file jamanetwopen-e2412050-s001.pdf]

Data Sharing Statement

Islam. Racial and Ethnic Inequities in Cancer Care Continuity During the COVID-19 Pandemic Among Those With SARS-CoV-2. *JAMA Netw Open*. Published May 20, 2024. doi:10.1001/jamanetworkopen.2024.12050

Data
Data available: No
